# The systematic and participatory development of a patient decision aid about terminal devices for people with upper limb absence: The PDA-TULA

**DOI:** 10.1097/PXR.0000000000000232

**Published:** 2023-03-20

**Authors:** Nienke Kerver, Laura Boerema, Michael A. H. Brouwers, Corry K. van der Sluis, Sacha van Twillert

**Affiliations:** 1 Department of Rehabilitation Medicine, University of Groningen, University Medical Center Groningen, Groningen, the Netherlands; 2 De Hoogstraat Rehabilitation, Utrecht, the Netherlands; 3 University of Groningen, University Medical Center Groningen, UMC Staff Policy and Management Support, Groningen, the Netherlands

**Keywords:** decision support techniques, artificial limbs, upper extremity, decision making, shared, amputation, traumatic, upper extremity deformities, congenital, cocreation, patient decision aid

## Abstract

Selecting an upper limb prosthesis seems to be a challenge considering the high rejection rates. A patient decision aid (PDA) could support the decision-making process by providing information about available options and clarifying the patients' values related to those options. This study aims to describe the developmental process of a PDA about terminal devices (TDs) for people with upper limb absence: PDA-TULA. The developmental process was based on The International Patient Decision Aid Standards. We aimed at adults with major unilateral upper limb absence. A steering group including patients, clinicians, researchers, software and implementation experts was composed. The content and design of the PDA were based on a qualitative literature meta-synthesis, focus groups with patients and clinicians, surveys among patients and prosthetists, a nationwide digital meeting with clinicians and prosthetists, and information from manufacturers. Information on features of TDs was systematically collected, ordered, and refined. Subsequently, drafts of the PDA-TULA were made, improved, integrated into the software, and alpha tested. The digital PDA-TULA consists of three parts: (1) information about TDs; (2) consideration of personal values regarding the TDs; (3) comparison of TD profiles with a personal profile based on indicated preferences. A summarizing overview is offered to patients and clinicians. To conclude, a digital PDA, which was integrated into the national working process of clinicians, was developed in a systematic co-creation process. The PDA enables patients and their significant others to consider and formulate their preferences about TDs during the prosthesis selection process.

## Background

The high rejection rates of upper limb prostheses (ULP) are associated with several reasons,^1-3^ among which discrepancies between perceived need and prostheses available.^1^ Considering the wide range of ULP (i.e., passive, cosmetic, body-powered, and myoelectric with 1 grip or multiple grips) and great variety in circumstances and preferences between individuals (e.g., appearance, control, and functionality),^4^ tailoring the prosthesis choice to their needs can be challenging.

Multiple studies suggested that a patient-centered ULP fitting approach may enhance prosthesis acceptance.^1-3,5^ However, in these studies, no tools to support such active patient involvement were offered yet. One tool that could support this role is a patient decision aid (PDA).^6^ Patient decision aids aim to improve the congruency of patients' choices with their preferences by providing information about the options and helping patients to find their values related to these options.^7^ Patients feel more knowledgeable and better informed about their values after using a PDA.^6^ Therefore, we developed a PDA with a primary focus on terminal devices for people with upper limb absence (TULA): the PDA-TULA. We defined a terminal device (TD) as the artificial replacement of the hand, excluding the prosthetic wrist and socket. This study aimed to describe the systematic and participatory developmental process of the PDA-TULA.

## Technique

The documentation template of the International Patient Decision Aid Standards was used to describe the key elements of the PDA developmental process.^8^

### Scope

**Figure 1.**
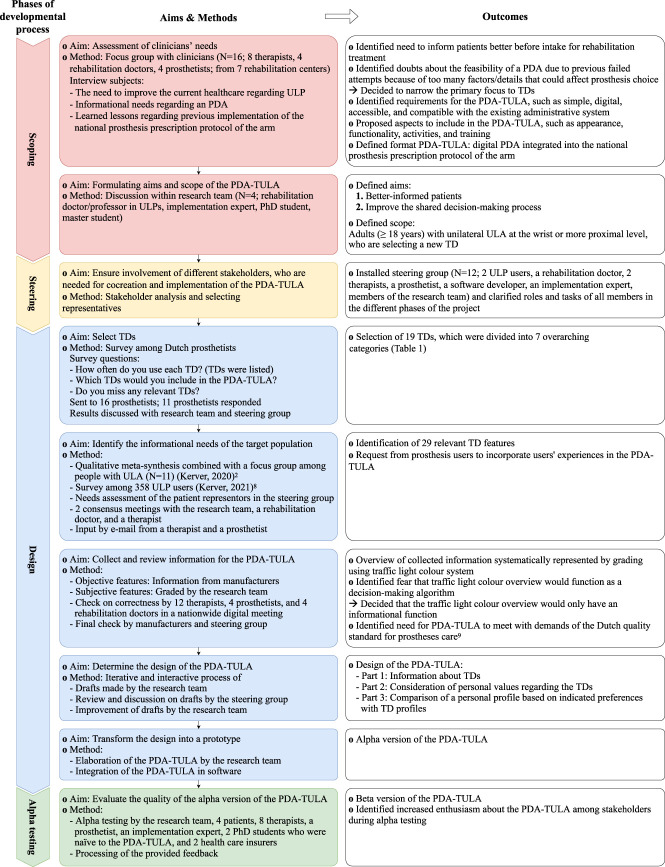
Overview of the developmental process of the PDA-TULA based on the International Patient Decision Aid Standards. PDA, patient decision aid; PDA-TULA, patient decision aid about terminal devices for people with upper limb absence; PPP-Arm, Prosthesis Prescription Protocol of the Arm; TD, terminal device; ULA, upper limb absence; ULP, upper limb prostheses.

Because of a previous unsuccessful attempt to develop a PDA, the importance of broad support, and the identification of eventual prerequisites, we first held an hour-long focus group with clinicians (Figure 1). All participating clinicians provided written informed consent. Based on their input, we decided to develop a digital PDA that was integrated into the national administrative system to prescribe prostheses: Prosthesis Prescription Protocol of the Arm.^9^ Subsequently, the aim of the PDA-TULA was formulated by the research team and confirmed by the steering group (see next paragraph): (1) to better inform patients about the available TDs and (2) to improve the shared decision-making process between patient and clinicians when selecting a TD. The PDA-TULA focuses on adults with unilateral ULA at the wrist or more proximal level, including both new and experienced prosthesis users.

### Steering group

To ensure that the needs of all involved parties were represented, a steering group with stakeholders was composed (Figure 1). This group provided input and feedback about the development, implementation, and evaluation of the PDA during 4 digital meetings and by e-mail.

### Design

First, we determined which information should be included in the PDA-TULA. Due to the great number of available TDs, it was impossible to include all. Therefore, prosthetists were questioned in a survey which TDs they used most or were deemed important to include in the PDA-TULA (Figure 1). They provided informed consent by filling out the survey. Based on survey results and discussions with the research team and steering group, TDs were selected for the PDA-TULA. To determine what aspects of those TDs we should include in the PDA-TULA, an extensive list of prosthetic features, possibly affecting prosthesis choice, was created based on the results of our previous studies.^4,10^ The list was shortened, reorganized, and refined during 2 consensus meetings, where factors that met full consensus were immediately included and factors that did not meet full consensus were discussed until consensus was met.

Next, we collected information about the features of these selected TDs (Figure 1). The outcomes of these features were graded on a traffic light color scale: red colors reflected unfavorable outcomes (e.g., heavy, costly), green colors favorable outcomes (e.g., light, cheap), and orange colors judgments in between red and green. Objective features (e.g., weight, grip force, wrist options, and harness needed) were collected from manufacturers' websites, manuals, or requested from manufacturers. Subjective features (e.g., lifelike appearance, reliability) were graded by the research team. The grading was discussed during a nationwide digital meeting with clinicians and prosthetists. Doubts about the function of this grading system as a decision-making algorithm arose and, consequently, the grading system would only have an informational function. Furthermore, the need for a PDA to comply with the Dutch quality standard for prostheses care was acknowledged.^11^ Last, the manufacturers and steering group had the opportunity to check the grading.

Subsequently, the information was translated into the PDA-TULA. This was a collaborative process in which the research team made drafts, received, and processed feedback from the steering group. This process was repeated until the steering group agreed about the design. Last, the PDA-TULA was integrated into the software, resulting in the alpha version.

### Alpha testing

The alpha version was tested by several stakeholders (Figure 1). Reactions were predominantly positive, e.g., the PDA provides clear information, looks good, and expectedly supports the prosthesis choice. Points of improvement mainly concerned software malfunctioning, phrasing, and provided information about the TDs. All feedback was processed, resulting in the beta version of the PDA-TULA. The beta testing will be described elsewhere.

## Results

In consecutive order, the digital PDA-TULA consists of a users' instruction, an explanation of aims and scope, a request to enter some personal information, followed by the 3 main parts: (1) information about TDs, (2) consideration of personal values regarding the TDs, and (3) comparing own preferences with features of the TDs. Within the Prosthesis Prescription Protocol of the Arm protocol, clinicians can send the patient an auto-generated e-mail containing login information.

### Part 1: Information about TDs

**Figure 2.**
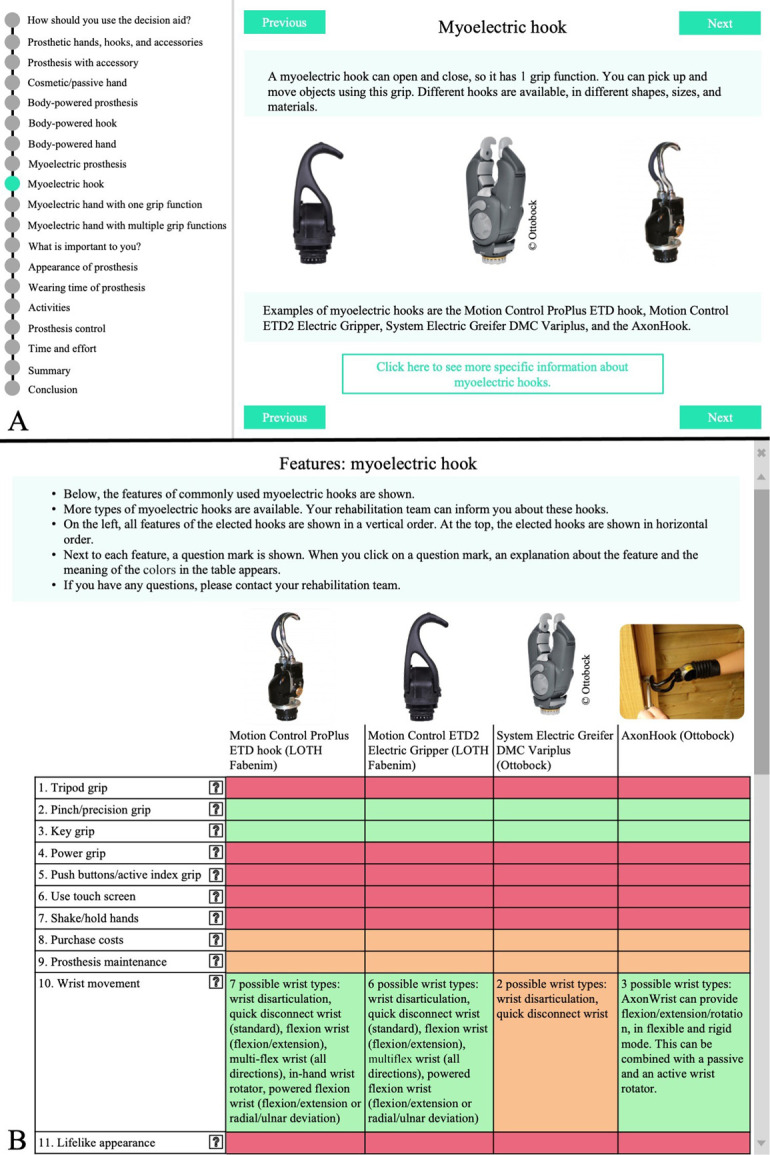
Examples of 2 pages from part 1 of the patient decision aid about terminal devices for people with upper limb absence. An information page about 1 category of TDs is depicted on page A. When clicking on “Click here to see more specific information about myoelectric hooks” page B appears, on which more detailed information about TDs belonging to the category is depicted. Features were graded on a traffic light color scale. When clicking on a question mark, additional information about the feature and the three-color scale appears. ETD, electric terminal device; TD, terminal device.

To avoid an overwhelming amount of information for new users, TDs are divided into 7 overarching categories on which general information is provided. To ensure enough details are available to interest experienced users, optionally, more detailed information about every TD is available by clicking on a dedicated link (Table 1; Figure 2); this included information regarding wrist options and the need for a harness. Furthermore, pictures of different sockets and TD types are shown in the PDA-TULA.

**Table 1.**
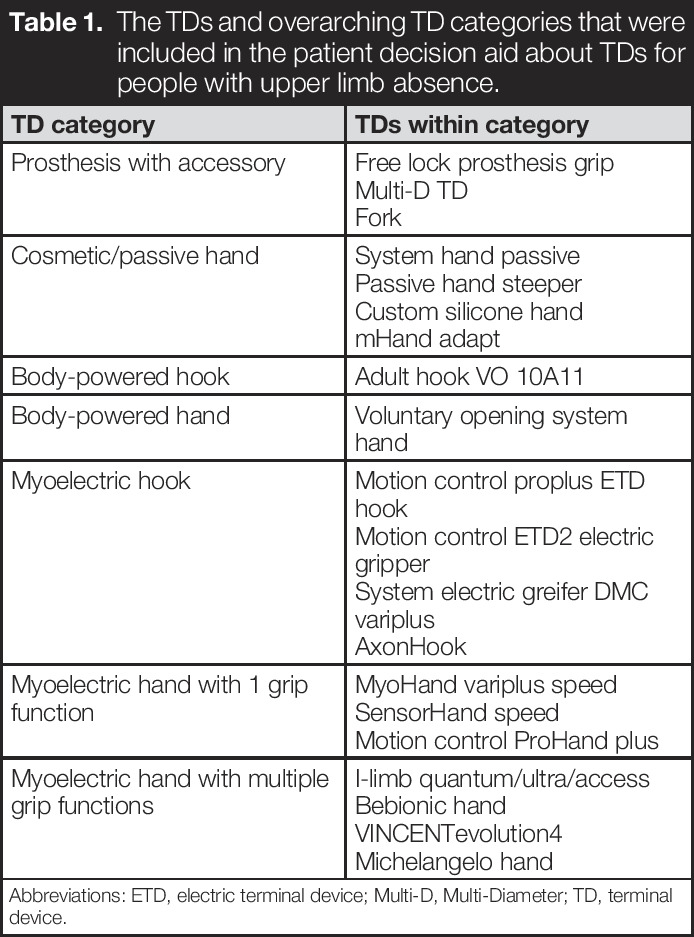
The TDs and overarching TD categories that were included in the patient decision aid about TDs for people with upper limb absence.

TD category	TDs within category
Prosthesis with accessory	Free lock prosthesis grip Multi-D TD Fork
Cosmetic/passive hand	System hand passive Passive hand steeper Custom silicone hand mHand adapt
Body-powered hook	Adult hook VO 10A11
Body-powered hand	Voluntary opening system hand
Myoelectric hook	Motion control proplus ETD hook Motion control ETD2 electric gripper System electric greifer DMC variplus AxonHook
Myoelectric hand with 1 grip function	MyoHand variplus speed SensorHand speed Motion control ProHand plus
Myoelectric hand with multiple grip functions	I-limb quantum/ultra/access Bebionic hand VINCENTevolution4 Michelangelo hand

Abbreviations: ETD, electric terminal device; Multi-D, Multi-Diameter; TD, terminal device.

### Part 2: Consideration of personal values regarding the TDs

**Figure 3.**
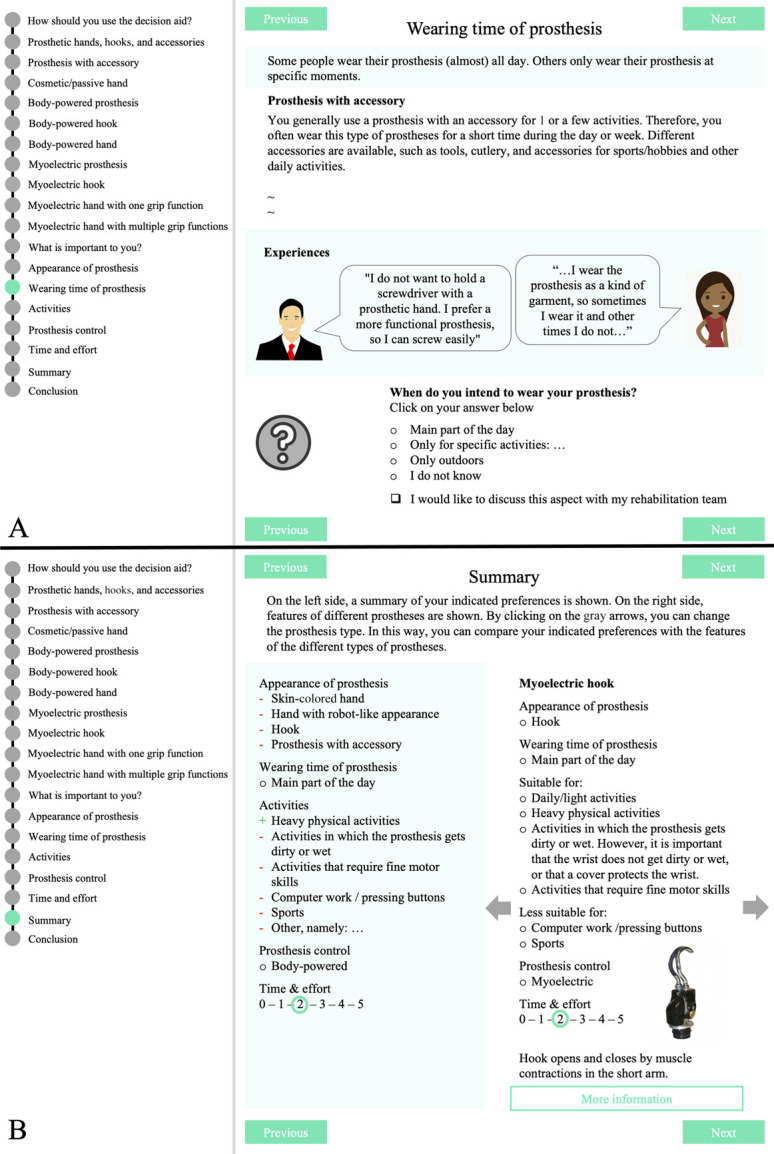
Examples of 2 pages from parts 2 and 3, respectively, of the PDA-TULA. (a) Shows information about the aspect “wearing time” with the corresponding question. A part of the information and pictures on this page were replaced by the “∼” signs. (b) Shows the summary page of the PDA-TULA. In green, the personal profile of the patient is shown. In white, the profile of one of the categories of TDs is shown. By clicking on one of the gray arrows, the profile of another TD category appears. PDA-TULA, PDA with a primary focus on terminal devices for people with upper limb absence; TD, terminal device.

The PDA-TULA intends to stimulate patients to consider what is important for them regarding their prosthesis and get aware of the consequences of certain choices. Therefore, 5 relevant prosthetic aspects, selected by the steering group, form the basis for consideration (Table 2). The advantages and disadvantages of the TD categories regarding a prosthetic aspect are first explained. Subsequently, patients are asked to provide their preference regarding this aspect (Figure 3(A)). Based on patient's feedback, the option to tick “I would like to discuss this aspect with my rehabilitation team” was added.

**Table 2.**
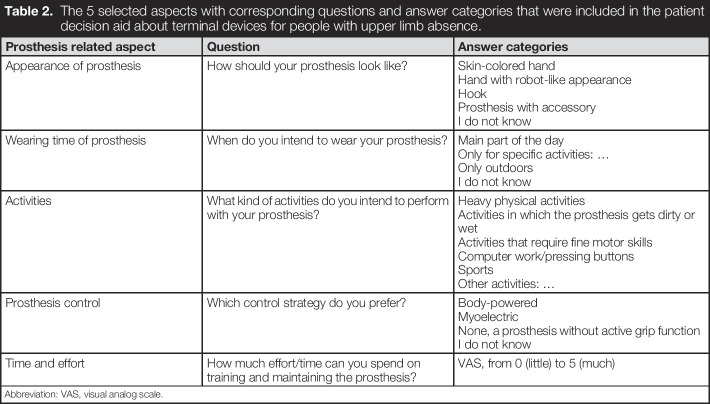
The 5 selected aspects with corresponding questions and answer categories that were included in the patient decision aid about terminal devices for people with upper limb absence.

Prosthesis related aspect	Question	Answer categories
Appearance of prosthesis	How should your prosthesis look like?	Skin-colored hand Hand with robot-like appearance Hook Prosthesis with accessory I do not know
Wearing time of prosthesis	When do you intend to wear your prosthesis?	Main part of the day Only for specific activities: … Only outdoors I do not know
Activities	What kind of activities do you intend to perform with your prosthesis?	Heavy physical activities Activities in which the prosthesis gets dirty or wet Activities that require fine motor skills Computer work/pressing buttons Sports Other activities: …
Prosthesis control	Which control strategy do you prefer?	Body-powered Myoelectric None, a prosthesis without active grip function I do not know
Time and effort	How much effort/time can you spend on training and maintaining the prosthesis?	VAS, from 0 (little) to 5 (much)

Abbreviation: VAS, visual analog scale.

### Part 3: Comparing own preferences with the categories of TDs

The PDA-TULA does not provide the final selection of a TD. No algorithms are available for such a selection, and the PDA-TULA would then be categorized as a Class II medical device according to European Medical Device Regulations with subsequent rules that should be followed.^12^ Instead, a personal profile is created based on the patient's indicated preferences. This profile can be compared with profiles of the TD categories (Figure 3(B)). Accordingly, the patient gets a sense of which TD suits his preferences best. Last, the patient can indicate his preferred TD category and specify questions for the next consultation. An overview summarizing the patient's and prosthetic profiles, preferred TD category, and patient's questions is generated to discuss prosthesis options with their clinicians.

## Discussion

The PDA-TULA offers potential benefits for patients and clinicians. First, the PDA-TULA enables patients to go through the information at their own pace at home and consider their preferences. Second, the generated summary encourages patients and clinicians to discuss the results of the PDA-TULA during their next consultation. This is important considering an often-found discrepancy between the patient's preferences and physicians' judgment.^13^ Last, the PDA-TULA enables all rehabilitation teams to use the same educational materials, which may lead to more unanimous prosthesis selection procedures across the country.

Some limitations regarding the PDA-TULA should be mentioned. First, the effectiveness of the PDA-TULA is not tested yet. Second, whether the PDA-TULA will result in higher prosthesis acceptance cannot be investigated because Dutch rejection rates before the introduction of the PDA-TULA are unknown. Third, for illiterate people, non-Dutch speakers, or people with low digital skills, assistance is needed to go through the PDA-TULA. Fourth, to keep the amount of information manageable, the PDA primarily focusses on TDs. To support the decision regarding, for instance, wrist options, harness, or socket options, separate PDAs or adding pages to the PDA-TULA might be considered in future. Last, because scientific evidence is lacking, and it was not feasible to collect data from a large group of patients, the subjective features in the traffic light overview were based on expert opinions. When more scientific evidence becomes available, the PDA-TULA should be updated using patient opinions.

## Conclusion

The PDA-TULA was developed in a systematic cocreation process and nationally integrated into the clinicians' working processes. The PDA-TULA enables patients and their significant others to consider their preferences and questions about TDs of the UL during the prosthesis selection process.

## Author contributions

N.K.: conceptualization, investigation, visualization, and writing—original draft. L.B.: investigation, visualization, and writing—original draft. M.A.H.B.: resources, writing—review and editing. C.K.v.d.S.: conceptualization, funding acquisition, project administration, resources, supervision, and writing—review and editing. S.v.T.: conceptualization, funding acquisition, supervision, and writing—review and editing. N.K. and L.B. contributed equally to this work.

## Ethics review and approval

The local Medical Ethics Review Board of the University Medical Center Groningen assessed the study protocol and decided to waive formal study approval (METc 2018/582).

## Clinical trial registration

This study is registered at the Netherlands Trial Register (www.trialregister.nl): NL7682.

## Funding

This study was funded by ZonMW (project number: 853001102). The funders had no role in the design of the PDA-TULA or the authoring of this clinical note.

## Declaration of conflicting interest

The authors disclosed no potential conflicts of interest with respect to the research, authorship, and/or publication of this article.

## Acknowledgments

The authors would like to thank the members from the steering group for their help with the development of the PDA-TULA: F. de Backer-Bes, prosthetist from De Hoogstraat Rehabilitation; H. Beijering, ULP user; C. Bootsman, ULP user; M. Heurman, therapist from Roessingh Center for Rehabilitation; E. de Jeu, implementation expert from Vilans; L. Thijssen, software developer from Isatis Projects; and P.A. Wijdenes, therapist from the University Medical Center Groningen. In addition, we would like to thank all clinicians who participated in the consensus meetings and the nationwide digital meeting. Last, we would like to thank all people who provided feedback on the alpha version of the PDA-TULA.

## Supplemental material

No supplemental digital content is available in this article.
